# Prevalence of physically active females at risk for the female athlete triad in Spain

**DOI:** 10.1080/15502783.2025.2590641

**Published:** 2025-12-02

**Authors:** Ana Torres Dos Ramos, Montse Bellver, Laura Esquius, Iván Martínez Pastor, Antonia Barea Montes, Ana Andrés

**Affiliations:** a Faculty of Health Sciences, Universitat Oberta de Catalunya, Barcelona, Spain; b Centro de Alto Rendimiento (CAR), Sant Cugat del Vallès, Barcelona, Spain; c Consorcio Sanitario de Terrassa, Terrassa, Spain; d Epi4health Research Group, Faculty of Health Sciences, Universitat Oberta de Catalunya, Barcelona, Spain; e Gesein, S.L., Madrid, Spain; f Faculty of Psychology, Education and Sport Sciences Blanquerna, Ramon Llull University, Barcelona, Spain

**Keywords:** Amenorrhea, disordered eating, energy deficiency, menstrual cycle, women in sport

## Abstract

**Background:**

The female athlete triad (Triad) refers to the interrelationship between energy availability, menstrual function, and bone health, which may have clinical and subclinical manifestations including eating disorders, functional hypothalamic amenorrhea, and osteoporosis. The aim of this study was to analyze the prevalence of physically active 15- to 45-year-old Spanish women at risk for the Triad.

**Methods:**

A descriptive cross-sectional study was carried out using the validated questionnaires ‘Low Energy Availability in Females Questionnaire’ (LEAF-Q) and the ‘Female Athlete Screening Tool’ (FAST). Exercising women were invited to participate anonymously and voluntarily by email via Spanish sports clubs and federations, as well as through social networks. The questionnaires were also distributed to elite athletes attending the High Performance Center of Sant Cugat del Vallès (Barcelona).

**Results:**

The study participants comprised 1154 physically active women (age = 28.5 ± 7.7 years; height = 165.2 ± 6.2 cm; body weight = 60.5 ± 9.7), representing 78 recreational and competitive level sports. The results showed that 40.0% (*n* = 462) were considered at risk for the Triad, while 24.3% (*n* = 280) were at risk for subclinical eating disorder, and 7.3% (*n* = 84) at risk for clinical eating disorder. Other results were: menstrual dysfunction displayed a positive correlation with the LEAF-Q score (r = 0.499, *p* = < 0.001); FAST presented an association with the LEAF-Q score (r = 0.252, *p* < 0.001); and body weight (r = 0.207, *p* = < 0.001) and body mass index (r = 0.225, *p* = < 0.001) displayed a positive correlation with the FAST score.

**Conclusion:**

Based on this research, the implementation of strategies for the prevention, diagnosis and treatment of the Triad disorders should be made a priority among physically active Spanish women.

## 1. Introduction

The female athlete triad (henceforth the Triad) is a syndrome of interrelated conditions observed in physically active girls and women that involves, whether in isolation or combined, low energy availability (LEA) with or without disordered eating (DE), menstrual dysfunction (MD), and compromised bone health [1,2]. According to its conceptual model, each component of the Triad exists along a continuum. At one end lie healthy states of optimal energy availability, eumenorrhea, and bone health; at the other, pathological endpoints, such as eating disorders (ED), functional hypothalamic amenorrhea (FHA), and osteoporosis. Each spectrum that makes up the Triad contemplates subclinical alterations, highlighting the presence of intermediate conditions such as luteal phase defects, anovulation, and low bone mineral density. Other clinical and physiological consequences of the Triad are endothelial dysfunction, altered lipid profile, transient infertility, failure to achieve peak bone mass, increased risk of bone stress injuries, and impaired physical performance, among others. Although the Relative Energy Deficiency in Sport (RED-S) model, introduced by the International Olympic Committee (IOC) in 2014, also addresses the effects of LEA in athletes, both models may coexist, advancing evidence-based medicine [3].

Energy availability is defined as the energy available for basic physiological functions resulting from the difference between dietary energy intake and exercise energy expenditure, normalized to fat-free mass (FFM) [4]. The LEA observed in physically active women has been positioned as a key etiological factor of the Triad, and can occur through four possible pathways: DE; ED; intentional weight loss (without DE); or inadvertent undereating [2]. In mammals, during periods of energy deficiency available metabolic fuel is distributed away from the expendable and energetically costly processes of reproduction, growth and fat storage to prioritize those processes essential for survival, including cell maintenance, thermoregulation and locomotion [5]. To facilitate energy conservation, a cascade of metabolic and energetic adaptations occurs, including the suppression of resting energy expenditure, decreased concentrations of the metabolic hormones total triiodothyronine, insulin-like growth factor-I, leptin, and insulin, and the upregulation of growth hormone and cortisol [6]. The adaptive response described above restores energy balance and body weight stability, while maintaining adverse health effects secondary to LEA [1,2,6].

Short-term experiments (4−5 days) conducted by Loucks et al [7]. established the conceptual model of a specific energy availability threshold of 30 kcal/kg FFM/day, below which a series of metabolic and endocrine adaptations were observed. Nevertheless, subsequent long-term studies discourage using absolute energy availability thresholds to prevent Triad sequelae. Although such a threshold increases the probability of experiencing MD by 50%, individual susceptibility varies significantly, influenced by factors such as psychogenic stress, gynecological age, low carbohydrate availability, daily hours in negative energy balance, or genetic predisposition to hypothalamic amenorrhea [4,7,8].

Sports that emphasize leanness or a low body weight have been associated with an increased risk of Triad disorders [1,9–11]. However, any exercising women may be susceptible to this condition, regardless of the type of sport they engage in. Additionally, compared to the general population, athletes have a higher risk of developing eating pathologies. Although the etiopathogenesis of ED is multifactorial, it has been suggested that the combination of sociocultural pressures and sport-specific expectations and demands may predispose athletes to developing ED or DE behaviors [10,12,13]. Type of sport, level of competition, pressures on weight, size and appearance from teammates, coaches and the sporting environment in general, as well as the display of athletes' bodies in competitive environments, judged not only on their performance but also their physical attractiveness, are risk factors for DE/ED [10,13]. Moreover, although men are also susceptible to the Triad, female athletes face greater sociocultural and sport-related pressures regarding body image, increasing their risk of eating pathologies [12].

The estimated prevalence of female athletes presenting all three Triad conditions simultaneously is relatively low (0%−15.9%), increasing to 2.7%−27.0% for two combined components and 16.0%−60.0% for any individual condition [9]. De Souza et al [14]. reported severe menstrual disturbances in 37.2% of exercising women's cycles, compared to no cases in sedentary women. Furthermore, subtle menstrual disturbances occurred in 50% of physically active women versus 4.2% in sedentary controls. Notably, these disturbances occurred despite regular cycle lengths (26−35 days), highlighting that cycle length alone is not an accurate marker of ovarian function in this population.

This study aimed to assess the prevalence of recreational and competitive physically active Spanish women at risk for the Triad, identifying the concomitant presence of clinical and subclinical ED risk. Additionally, the possible relationship between the risk of Triad disorders and age, level of participation and sporting activity engaged in was evaluated, aligning with previous hypotheses.

## 2. Methods

### 2.1. Study design

A descriptive cross-sectional study was conducted using an anonymous and voluntary online questionnaire, conducted ad-hoc to determine the risk of the Triad in physically active Spanish women, after approval by the Ethics Committee of the Universitat Oberta de Catalunya (CE22-TF07). The study protocols and procedures were developed in accordance with the standards outlined in the Declaration of Helsinki. All participants provided written informed consent before participating in the study. Data collection was performed over a three-month period (April 2022 to June 2022). No incentives of any kind were offered.

### 2.2. Participants

Physically active Spanish women aged between 15 and 45 years were invited to participate via email -10783 emails were sent to Spanish sports clubs and federations to this end- and through social networks. Additionally, the questionnaire was distributed to elite athletes attending the High Performance Center of Sant Cugat del Vallès (Barcelona), through the Terrassa Health Consortium (CAR-CST). The eligibility criteria for the study were 1) being female; 2) being aged between 15 and 45 years; 3) being resident in Spain; 4) doing ≥3 h/week of intentional exercise; and 5) not being pregnant.

### 2.3. Questionnaire data

Sociodemographic information was collected, including weight (kg), height (cm), diagnosed diseases and associated medication, as well as data related to sporting activity: sporting activity engaged in, weekly training hours and level of sports participation (recreational, regional, national, or international). Participants were then asked to fill out the Low Energy Availability in Females Questionnaire (LEAF-Q) and Female Athlete Screening Tool (FAST) questionnaires.

### 2.4. Low Energy Availability in Females Questionnaire (LEAF-Q)

The LEAF-Q was designed to identify female athletes at risk for the Triad by assessing current LEA and/or MD and/or low bone mineral density [15]. It is a screening tool that has been validated in endurance athletes (78% sensitivity, 90% specificity) and assesses self-reported physiological symptoms associated with persistent energy deficiency. It comprises 25 items related to injury history, gastrointestinal function, and reproductive function. The cut-off points proposed are as follows: ≥2 for injuries; ≥2 for gastrointestinal symptoms; and ≥4 for MD; with scores of ≥8 considered at risk for the Triad. It is recommended that the LEAF-Q be used in combination with a validated DE/ED screening tool. In line with a previous study [16], participants were considered to have MD if they reported <9 menstrual cycles per year and/or the absence of >3 consecutive menstrual cycles. Prior MD was defined as primary amenorrhea (menarche at age ≥15 years) or secondary amenorrhea (absence of >3 consecutive menstrual cycles) [16,17].

### 2.5. Female Athlete Screening Tool (FAST)

Developed and validated as a screening tool for identifying female athletes at risk for ED, FAST has demonstrated high internal consistency (Cronbach’s *α* = 0.87), discriminant validity, and concurrent validity compared to other validated psychometric tools that identify eating pathologies among the general population (EDE-Q and EDI−2) [18]. This 33-item questionnaire is scored on a 4-point Likert-type scale, assigning decreasing values ​​based on the response (4 points = frequently; 3 points = sometimes; 2 points = rarely; 1 point = never), with a reverse system for questions 15, 28, and 32. The result is a risk score of between 33 (low risk) and 130 (high risk). A score of 77–94 is considered subclinical risk, and a score of >94 indicates a clinical ED risk.

### 2.6. Data analysis

The statistical analysis was performed using IBM SPSS® Statistics for Mac OS, version 29.0.2.0. Categorical variables were described using frequencies and percentages. Continuous data were expressed as mean values ​​(standard deviations [ ± ]). Data were not adjusted to normality according to the Kolmogorov-Smirnov test. Significant differences between groups of continuous variables were identified using the nonparametric Mann-Whitney U test for independent variables or the Kruskal Wallis test, as appropriate. Differences between Triad/ED risk groups and categorical variables of interest, such as sports category, were analyzed using the chi-square test. Spearman's correlation (r) was applied to analyze the relationship between quantitative variables. The level of statistical significance was set at *p* < 0.05.

Women using hormone therapy were excluded from LEAF-Q menstrual scoring, with only the injury and gastrointestinal function subcategories being considered.

## 3. Results

### 3.1. Participant characteristics

**Figure 1.**
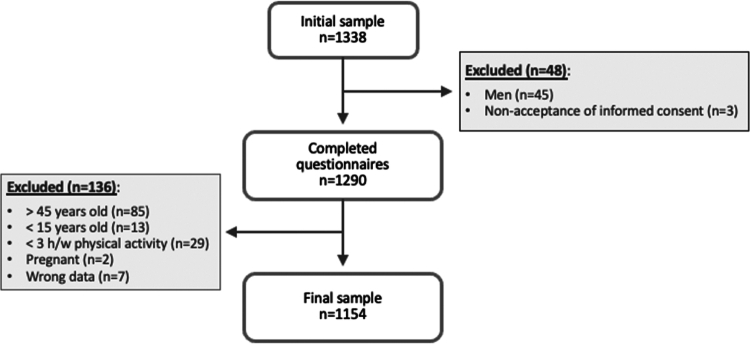
Study flowchart.

A total of 1338 responses were received (Figure 1). Of these, 184 were excluded for the following reasons: non-acceptance of informed consent (*n* = 3); non-compliance with inclusion criteria (*n* = 174); and questionnaire with erroneous data (*n* = 7). The final analysis therefore comprised a total of 1154 physically active women [age = 28.5 (±7.7), height = 165.2 cm (±6.2 cm), body weight = 60.5 (±9.7), body mass index (BMI) = 22.1 kg/m2 (±3.1 kg/m2)], representing 78 sports (Table 1). In line with previous studies [10], these groups were subsequently categorized into seven types: technical, endurance, esthetic, weight category, ball game, power, and antigravitation. Additionally, sporting activities were categorized as leanness (endurance, esthetic, weight category, anti-gravitation) or non-leanness sports (technical, ball game, power). Participants were grouped according to sports participation level. Finally, 274 athletes (23.7%) were excluded from the LEAF-Q menstrual function subcategory due to hormonal contraceptive use.

**Table 1.** Descriptive analysis of sample by type of sport according to competitive level.

		Recreational (50.8%)	Regional (23.8%)	National (19.2%)	International (6.2%)
**Type of sporting activity**					
Technical	19 (1.6%)	6 (31.6%)	2 (10.5%)	5 (26.3%)	6 (31.6%)
Endurance	389 (33.7%)	224 (57.6%)	71 (18.3%)	70 (18.0%)	24 (6.2%)
Esthetic	55 (4,8%)	12 (21.8%)	10 (18.2%)	19 (34.5%)	14 (25.5%)
Weight category	28 (2.4%)	13 (46.4%)	5 (17.9%)	9 (32.1%)	1 (3.6%)
Ball sport	324 (28.1%)	62 (19.1%)	162 (50.0%)	82 (25.3%)	18 (5.6%)
Power	293 (25.4%)	237 (80.9%)	21 (7.2%)	31 (10.6%)	4 (1.4%)
Antigravitation	46 (4.0%)	32 (69.6%)	4 (8.7%)	6 (13.0%)	4 (8.7%)

### 3.2. LEAF-Q scores

For this questionnaire, 40.0% of exercising women (*n* = 462) had a score consistent with Triad risk (Table 2). The mean total LEAF-Q score was 7.05 ± 4.33. The mean subcategory scores were: 1.66 ± 2.03 for injuries; 2.65 ± 2.25 for gastrointestinal function; and 2.74 ± 2.51 for reproductive function. For the subcategories, 47%, 63%, and 33% scored above the LEAF-Q cutoff for injuries, gastrointestinal symptoms, and MD, respectively. No differences were found between groups associated with height, age, or level of sports participation. At-risk Triad participants reported significantly lower weight and BMI than those at low risk according to the questionnaire (59.4 kg ± 8.3 kg vs. 61.2 ± 10.5 kg, *p* = 0.035 and 21.7 ± 2.5 vs. 22.4 ± 3.4, *p* = 0.029, respectively). Exercising women at risk for the Triad trained more hours than the low-risk group (9.0 ± 5.8 vs. 8.3 ± 5.7, *p* = 0.003). Very weak, although statistically significant, correlations were observed between LEAF-Q scores and body weight (r = -0.055, *p* = 0.035), BMI (r = -0.070, *p* = 0.029), weekly training hours (r = 0.100, *p* = 0.003), and sporting category (r = -0.135, *p* = < 0.001).

**Table 2.** Descriptive analysis of all participants stratified into groups based on total LEAF-Q score.

LEAF-Q				
		Low-risk (*n* = 692)	At-risk (*n* = 462)	
	All (*n* = 1154)	<8	≥8	*p*-value
Height (cm)	165.2 ± 6.2	165.3 ± 6.4	165.1 ± 6.1	.904
Weight (kg)	60.5 ± 9.7	61.2 ± 10.5	59.4 ± 8.3	.035*
BMI (kg/m^2^)	22.1 ± 3.1	22.4 ± 3.4	21.7 ± 2.5	.029*
Age (year)	28.5 ± 7.7	28.5 ± 7.8	28.3 ± 7.6	.657
**Age group**				.806
15−24	401 (34.7%)	242 (60.3%)	159 (39.7%)	
25−34	487 (42.2%)	287 (58.9%)	200 (41.1%)	
35−45	266 (23.1%)	163 (61.3%)	103 (38.7%)	
Weekly training (hours)	8.6 ± 5.7	8.3 ± 5.7	9.0 ± 5.8	.003*
**Sports participation level**				.615
Recreational	586 (50.8%)	348 (59.4%)	238 (40.6%)	
Regional	275 (23.8%)	174 (63.3%)	101 (36.7%)	
National	222 (19.2%)	129 (58.1%)	93 (41.9%)	
International	71 (6.2%)	41 (57.7%)	30 (42.3%)	
**Sporting category**				<.001*
Technical	19 (1.6%)	11 (57.9%)	8 (42.1%)	
Endurance	389 (33.7%)	197 (50.6%)	192 (49.4%)	
Esthetic	55 (4,8%)	34 (61.8%)	21 (38.2%)	
Weight category	28 (2.4%)	15 (53.6%)	13 (46.4%)	
Ball sport	324 (28.1%)	221 (68.2%)	103 (31.8%)	
Power	293 (25.4%)	185 (63.1%)	108 (36.9%)	
Antigravitation	46 (4.0%)	29 (63.0%)	17 (37.0%)	
**Risk sport category**				<.001*
Non-leanness sports	636 (55.1%)	417 (65.6%)	219 (34.4%)	
Leanness sports	518 (44.9%)	275 (53.1%)	243 (46.9%)	

* *p* < 0.05.

Values are displayed as mean ± SD or numbers with percentages. Abbreviations: BMI, body mass index; LEA, low energy availability.

### 3.3. FAST questionnaire scores

A total of 280 physically active women (24.3%) were identified as being at risk for subclinical ED (score 77−94) from the results obtained. A further 84 participants (7.3%) were identified as being at risk for clinical ED according to the cut-off point >94 on the FAST questionnaire (Table 3). No significant differences were found with respect to height, age group, sports participation level, or sporting category. Differences between groups were observed in the variables body weight and BMI (*p* < 0.001), as well as in the number of hours of weekly training (*p* < 0.013). The risk of ED increased inversely with age, showing a higher risk in younger women (*p* < 0.048). Grouping by risk sporting category (leanness/non-leanness sports) yielded differences between groups (*p* = 0.028). Very weak but statistically significant correlations were observed between FAST score and age (r = -0.057, *p* = 0.048) and weekly training hours (r = 0.065, *p* = 0.013). Body weight (r = 0.207, *p* < 0.001) and BMI (r = 0.225, *p* < 0.001) presented a weak and statistically significant correlation associated with the total score from the questionnaire.

**Table 3.** Descriptive analysis of all participants stratified into groups based on total FAST score.

FAST					
		Healthy score (*n* = 790)	Subclinical score (*n* = 280)	Clinical score (*n* = 84)	
	All (*n* = 1154)	<77	77−94	>94	*p*-value
Height (cm)	165.2 ± 6.2	165.1 ± 6.2	165.3 ± 6.5	166.2 ± 5.5	.235
Weight (kg)	60.5 ± 9.7	59.8 ± 9.7	62.1 ± 10.0	61.4 ± 7.4	<.001*
BMI (kg/m^2^)	22.1 ± 3.1	21.9 ± 3.1	22.7 ± 3.2	22.2 ± 2.6	<.001*
Age (year)	28.5 ± 7.7	28.7 ± 7.7	28.2 ± 8.0	26.6 ± 7.2	.048*
**Age group**					.059
15−24	401 (34.7%)	261 (65.1%)	102 (25.4%)	38 (9.5%)	
25−34	487 (42.2%)	347 (71.3%)	106 (21.8%)	34 (7.0%)	
35−45	266 (23.1%)	182 (68.4%)	72 (27.1%)	12(4.5%)	
Weekly training (hours)	8.6 ± 5.7	8.5 ± 5.8	8.6 ± 5.2	10.1 ± 6.5	.013*
**Sports participation level**					.134
Recreational	586 (50.8%)	394 (67.2%)	145 (24.7%)	47 (8.0%)	
Regional	275 (23.8%)	198 (72.0%)	60 (21.8%)	17 (6.2%)	
National	222 (19.2%)	141 (63.5%)	63 (28.4%)	18 (8.1%)	
International	71 (6.2%)	57 (80.3%)	12 (16.9%)	2 (2.8%)	
**Sporting category**					.147
Technical	19 (1.6%)	14 (73.7%)	5 (26.3%)	0 (0%)	
Endurance	389 (33.7%)	257 (66.1%)	93 (23.9%)	39 (10.0%)	
Esthetic	55 (4,8%)	31 (56.4%)	20 (36.4%)	4 (7.3%)	
Weight category	28 (2.4%)	17 (60.7%)	8 (28.6%)	3 (10.7%)	
Ball sport	324 (28.1%)	234 (72.2%)	73 (22.5%)	17 (5.2%)	
Power	293 (25.4%)	201 (68.6%)	74 (25.3%)	18 (6.1%)	
Antigravitation	46 (4.0%)	36 (78.3%)	7 (15.2%)	3 (6.5%)	
**Risk sport category**					.028*
Non-leanness sports	636 (55.1%)	449 (70.6%)	152 (23.9%)	35 (5.5%)	
Leanness sports	518 (44.9%)	341 (65.8%)	128 (24.7%)	49 (9.5%)	

* *p* < 0.05. Values are displayed as mean ± SD or numbers with percentages. Abbreviations: BMI, body mass index; LEA, low energy availability.

### 3.4. LEAF-Q and FAST questionnaire scores

Physically active women at risk for subclinical and clinical ED were at higher risk of the Triad (LEAF-Q total score ≥8) than participants with a healthy FAST score (45.4% vs. 35.4% and 65.5% vs. 35.4%, respectively; Tables 4 and 5). The group of women at clinical risk of ED were 3.08 times more likely to be at risk for the Triad based on the LEAF-Q score (OR = 3.08, 95% CI = 1.94−4.93, *p* < 0.001). A weak, although statistically significant, correlation was observed between the FAST and LEAF-Q scores (r = 0.252, *p* < 0.001).

**Table 4.** Cross-tabulation of FAST and LEAF-Q score categories.

LEAF-Q				
		Low-risk (*n* = 692)	At-risk (*n* = 462)	
FAST risk	All (*n* = 1154)	<8	≥8	*p*-value
				<.001*
Healthy score	790 (68.5%)	510 (64.6%)	280 (35.4%)	
Subclinical	280 (24.3%)	153 (54.6%)	127 (45.4%)	
Clinical	84 (7.3%)	29 (34.5%)	55 (65.5%)	

* *p* < 0.05.

### 3.5. Menstrual function

With regard to menstrual function, 39.1% (*n* = 451) of the total sample (*n* = 1154) reported previous MD (11.0% primary amenorrhea, 28.1% secondary amenorrhea), 7.8% (*n* = 90) did not know if their menstrual cycle was healthy, 28.6% (*n* = 330) had had problems with heavy bleeding in the past, and 32.1% (*n* = 371) had experienced changes in their menstrual cycle after increasing the intensity, frequency, or duration of physical exercise. Of the total, 23.7% (*n* = 274) reported having used hormonal contraceptives, 15.4% (*n* = 178) of which had used combined oral contraceptives for the following reasons: 32% contraception; 19.7% reduction of pain associated with menstruation; 19.1% regulation of menstrual cycle; 11.8% amenorrhea; 5.6% reduction of menstrual bleeding; 5.1% polycystic ovary syndrome (PCOS); 3.9% endometriosis; and 2.8% other reasons. As for other non-oral hormonal contraceptives (e.g. intrauterine device), 96 participants reported having used these. Finally, five exercising women had received hormonal contraceptives in the absence of menarche, while two participants had never menstruated and used no hormonal contraceptives.

The menstrual status of participants not using hormonal contraceptives (*n* = 880) is presented in Tables 6 and 7. Menstrual abnormalities reported by women at Triad risk were significantly more prevalent than among women whose LEAF-Q score was consistent with low risk (OR = 7.371, 95% CI = 5.36–10.14, *p* < 0.001). Participants at clinical risk of ED were twice as likely to have MD based on their FAST risk score (10.7% vs. 5.3%, *p* = 0.012). The MD showed a statistically significant and moderate correlation with the LEAF-Q score (r = 0.499, *p* = < 0.001). The correlation between the score obtained in the FAST questionnaire and menstrual disorders was significant but very weak (r = 0.115, *p* = < 0.001).

**Table 5.** Cross-tabulation of LEAF-Q and FAST score categories.

FAST					
	All (*n* = 1154)	Healthy score (*n* = 790)	Subclinical score (*n* = 280)	Clinical score (*n* = 84)	
LEAF-Q risk		<77	77−94	>94	*p*-value
					<.001*
Low-risk	692 (60.0%)	510 (73.7%)	153 (22.1%)	29 (4.2%)	
At-risk	462 (40.0%)	280 (60.6%)	127 (27.5%)	55 (11.9%)	

* *p* < 0.05.

**Table 6.** Self-reported menstrual function divided into groups based on LEAF-Q score category (*n* = 880).

LEAF-Q				
		Low-risk (*n* = 522)	At-risk (*n* = 358)	
Menstrual status	All (*n* = 880)	<8	≥8	*p*-value
				<.001*
Eumenorrhea	600 (68.2%)	444 (74.0%)	156 (26.0%)	
Dysfunction	280 (31.8%)	78 (27.9%)	202 (72.1%)	

* *p* < 0.05.

## 4. Discussion

The aim of the present study was to examine the prevalence of recreational and competitive physically active Spanish women at risk for the Triad, identifying the concomitant presence of clinical and subclinical ED risk. It is the first study to analyze the risk of the female athlete triad in Spain. The cohort of this study was composed of a representative sample of 1154 recreational and competitive exercising women from a wide range of sports. A total of 40.0% of the participants were considered at risk for the Triad based on LEAF-Q scores, a result similar to those of other studies, which have reported estimates of 40%−45% [19–21]. However, considerable heterogeneity is observed in the results reported by previous studies using LEAF-Q across a wide range of sports and competitive levels, with at-risk women ranging from 3% to 65% in different samples [16,22,23]. Although LEAF-Q was validated in endurance athletes, subsequent studies support its application to other sporting activities [15,19]. Recently, its suitability as a screening tool has also been analyzed in populations for which it has not been validated [22,23]. In this regard, Dasa et al. [22] reported suboptimal performance in identifying clinical symptoms of the Triad in soccer players, with the exception of MD. Rogers et al. [23] reported that injury and menstrual function subscale scores identified low bone mineral density and MD with a sensitivity of 100% and 80%, respectively. The gastrointestinal subscale cutoff failed to detect LEA markers. Despite its low specificity, it had high negative predictive value for low-risk screening.

Of those participants at risk for the Triad, 60.6% were not at risk for clinical or subclinical ED. This finding is consistent with current evidence, since LEA can be inadvertent or associated with intentional weight loss, without the presence of an eating pathology [1,15]. In line with this observation, we also found a higher weekly training load to be associated with an increased risk of the Triad, aligning with previous studies [16,19,21]. Evidence suggests that dietary restriction increases hunger, unlike exercise-induced energy deficits [1]. In contrast to previous research [19], LEAF-Q scores showed no competitive-level differences. Although Triad risks in elite athletes are well-documented, emerging evidence highlights high LEA, MD, and/or ED prevalence in recreational populations [14,16,21].

Furthermore, of those participants at risk for the Triad, 39.4% had concomitant risk of ED (27.5% subclinical, 11.9% clinical), with an association being found between questionnaires. Consistent with the findings of Logue et al. [19], in the present study statistically significant differences were observed between groups in their FAST score associated with weekly training hours. Excessive exercise can be linked to risk behaviors associated with DE, such as fasting, binge-eating, or self-induced vomiting [1,24]. Consequently, depending on the symptoms and severity of DE/ED, athletes may present a wide range of body weights [16]. In line with previous studies [10,20], exercising women with a higher body weight and BMI had an increased risk of clinical and subclinical ED. Interestingly, the average BMI was within the normal reference range for all groups, suggesting that many women with Triad/ED symptoms have an apparently healthy body mass [13,16,24]. Toro et al. [24] reported that approximately half of athletes with normal or below-average BMI perceived themselves as fat and expressed concerns about their weight or body shape. Exposure of the body in public was strongly associated with amenorrhea, ED symptoms, and risk attitudes and behaviors. Additionally, pressure exerted by the coach regarding participants’ eating, physical appearance, weight, and performance was significantly associated with bulimia. In this regard, Ceballos et al. [12] explored the risk beliefs, attitudes, and behaviors of 127 Spanish coaches regarding food and body, as well as the pressures they exert on their athletes. The results showed moderate levels of risk beliefs and weight pressure, with 70.2% reporting that weight gain reduces performance and 37.6% that their athletes were better if they weighed less, while only 27% did not engage in weight control practices.

Regarding the prevalence of subclinical and clinical symptoms of ED, the scores were 24.3% and 7.3%, respectively, similar to that reported by Folscher et al. [20] using the FAST questionnaire (26.8% and 5.2%, respectively). Compared to 5% in the general population [24], the prevalence of subclinical eating pathologies and manifestations of altered behaviors among female athletes is 20%−62%, and 6%−45% for clinical ED [13]. Although it has been identified as a potential risk factor for ED^18^, we did not observe significant differences between sports participation levels. In a meta-analysis, Chapa et al. [11] reported that type of sport was associated with higher levels of ED psychopathology, with no differences related to the competitive level. In line with this, our findings showed differences in grouping by sport risk category (leanness/non-leanness sports). Sports focused on leanness, esthetic appearance, and weight control have been recurrently associated with an increased risk of eating pathologies [1,9–11]. Nevertheless, high pressures associated with size, shape, and weight, as well as a high concern about body weight and appearance, have been reported in women who participate in sports not considered high risk [9,13,16]. The high prevalence of eating pathologies in non-leanness sports challenges the utility of risk classifications based exclusively on sporting activity, underscoring the need to prioritize individual, environmental, and sociocultural factors [13]. In contrast to previous research [11], higher FAST scores were associated with younger age, suggesting an elevated risk of ED in this population. Supporting this, Borowiec et al. [25] reported an increased risk of ED in adolescent athletes, identifying lower body satisfaction and participation in lean nonesthetic sports as predisposing factors.

In our study, participants who were at risk for the Triad and/or clinical ED exhibited a higher prevalence of menstrual disorders, in line with previous studies [21,23]. The observed association between LEAF-Q score and the presence of MD evidences the adverse effects of LEA on reproductive function [19]. Consistent with Sánchez et al. [26], women had difficulties in assessing their menstrual cycles and abnormal bleeding patterns. These results highlight the urgent need to implement menstrual education programs for the population of both sexes, especially at younger ages. It is essential that healthcare personnel assess the menstrual cycle as a vital sign, including pre-season screenings, to detect health issues early [17]. Additionally, while menstrual disorders in athletes often result from LEA-induced hypothalamic inhibition, they may be associated with other causes, such as hyperandrogenism [27]. Menstrual irregularity and hyperandrogenism are clinical features of PCOS, which may coexist with FHA, underscoring the importance of differential diagnosis [15,27].

More than one-third of the participants reported that the main reason for using contraception was amenorrhea, pain, heavy menstrual bleeding, or “cycle regulation.” Although prescription of combined oral contraceptives is common in women with FHA, it is important to emphasize that hormone therapy only creates an exogenous ovarian steroid environment that provides a false sense of security when induced withdrawal bleeding occurs [2]. Hormonal contraceptives do not restore spontaneous menstruation, and its resumption is essential as a reflection of the general health status of adolescent and adult women [17]. Furthermore, the heavy menstrual bleeding reported by some study participants may be a consequence of anovulatory cycles, which are very prevalent in physically active women [14,17]. Current evidence does not support the use of combined oral contraceptives as a treatment in women with FHA, as it is not associated with an improvement in bone mineral density and may even further compromise bone health as a result of first-pass effects on hepatic production of insulin like-growth factor−1 [2]. Therefore, nutritional intervention is prioritized as first-line treatment to resume menstruation in exercising women with energy deficiency-related amenorrhea. The adoption of a biopsychosocial focus by an interdisciplinary team will ensure an approach that is adapted to the etiology underlying LEA.

In summary, our findings indicate a concerning prevalence of Triad/ED risk among physically active Spanish women. These results highlight the need for more research specifically involving female participants. Given that current sport and exercise science guidelines are largely based on studies conducted with male samples [28], the applicability of existing evidence to women is limited due to the anatomical, physiological, and endocrinological differences between sexes. Therefore, future investigations focusing on female athletes are warranted to enhance understanding of women’s physiology and to inform evidence-based strategies aimed at improving their health and performance.

### 4.1. Limitations

Self-reported measures may present recall bias and reporting bias, as they depend on the participants' interpretation, honesty, and ability to remember data [16]. Also, Triad risk was assessed using a screening tool validated only in endurance athletes, which could introduce bias in certain sports [15,22]. The absence of objective physiological assessments does not allow us to confirm the prevalence of Triad disorders. Additionally**,** the literature emphasizes the importance of considering body composition in terms of sports performance and optimal health of female athletes, not BMI or weight [13]. However, study limitations necessitated self-reported weight/height, though BMI remains useful to flag low-weight risks like LEA or DE behaviors [2,13]. Finally, 23.7% of participants were using hormonal contraceptives, preventing LEAF-Q menstrual assessment and likely underestimating Triad risk.

**Table 7.** Self-reported menstrual function divided into groups based on FAST score category (*n* = 880).

FAST					
		Healthy score (*n* = 613)	Subclinical score (*n* = 205)	Clinical score (*n* = 62)	
Menstrual status	All (*n* = 880)	<77	77−94	>94	*p*-value
					.012*
Eumenorrhea	600 (68.2%)	429 (71.5%)	139 (23.2%)	32 (5.3%)	
Dysfunction	280 (31.8%)	184 (65.7%)	66 (23.6%)	30 (10.7%)	

* *p* < 0.05.
